# NEWS

**DOI:** 10.7189/jogh.04.010201

**Published:** 2014-06

## AFRICA

• In a recent interview, Mr Andris Piebalgs, a member of the UN high–level panel advising on the post–2015 agenda, gave his thoughts on tackling the global crisis of youth unemployment. He highlighted the link between work opportunities and social disruption, saying that the main group of concern is young people in sub–Saharan Africa, with increased expectations and decreased opportunities. In the developed world, the youth unemployed have social protection – lacked by those in developing countries. However, he believes in similar solutions: good primary education; more liberalised and competitive economies; increased trade; and strengthened private sectors. He believes that health, nutrition and the agricultural sector are key to tackling youth unemployment in developing countries, and civil society programmes are important catalysts for getting young people into employment. (*The Guardian*, 14 Nov 2013)

• Nelson Mandela’s life, spanning 1918–2013, was defined by courage and a commitment to inclusiveness and equality. His associations with global health are broad and deep, including chairing the Board of the Vaccine Fund, which mobilized resources for the Global Alliance for Vaccines and Immunization. However, his contribution to the battle against AIDS is his greatest global health legacy; he viewed it as the urgent struggle for a new generation, which disproportionately affected the poor in Africa. His fundraising for HIV/AIDS and advocacy work to promote health care and reduce stigma means that his loss is felt particularly strongly in this group. He made human rights central to global political discourse, and moves towards universal health coverage and sustainable development goals owe much to his vision and insight. (*The Lancet*, 9 Dec 2013)

• The first mass vaccination campaign in Africa with a vaccine that does not need constant refrigeration provided complete coverage, and the vaccine stayed viable in temperatures up to 39°C. The meningitis A campaign in Benin is a breakthrough for both the vaccine (MenAfrVac®) and for increasing the efficiency, coverage and affordability of other vaccines, especially in remote areas where it is difficult to keep vaccines cold. It could reduce the number of under–vaccinated children and the cost of vaccine administration. The vaccine was developed through the PATH–WHO Meningitis Vaccine Project using a development model to provide an effective, affordable and long–term solution to epidemic meningitis in parts of Africa, which has killed or disabled thousands of people over the years. (*PATH,* 19 Feb 2014)

• Malawi has one of the highest rates of maternal mortality in the world, and Chief Kwataine, headman of the district of Ntcheu, attributes the root of the problem to a culture of secrecy where sex, pregnancy and childbirth are taboo subjects. This meant that pregnant women were not talking to doctors and nurses, and not accessing the care they needed. He came up with the idea of ‘secret mothers’ – female elders, respected in their communities – who could break through this barrier to support and advise pregnant women in their communities. The programme seems to have saved lives in the district, and Chief Kwataine is trying to spread it to other villages. (*Public International Radio*, 23 Feb 2014)

• The first Ebola outbreak in Guinea, West Africa, has killed at least 59 people and may be spreading to other countries. Guinea’s Health Ministry said that most of the known cases were in border areas near Sierra Leone and Liberia. The outbreak is of the Zaire strain, which has a 90% mortality rate and there is no known cure. Bats are the virus’ natural reservoir, and once humans are infected it readily spreads via bodily fluids. Outbreaks are contained by isolating the ill, and making sure that those treating them wear suitable protective clothing. Education is also crucial to prevent people panicking and fleeing, thus further spreading the outbreak. Médecins Sans Frontičres and WHO are working with the Health Ministry to contain the outbreak: the countries threatened by the disease are amongst the world’s poorest and cannot mount large public health efforts by themselves. (*New York Times*, 24 Mar 2014)

## ASIA

• In 2011, developing countries lost nearly US$ 1 trillion to fraud and corruption, more than received in foreign aid – and it is growing, warns Global Financial Integrity (GFI), a group that exposes financial corruption. The Middle East and North Africa saw the largest increases in the proceeds from illicit business, crime and corruption, followed by sub–Saharan Africa. Asia lost the largest amount of money, but when outflows are measured as a percentage of annual growth, sub–Saharan Africa has the biggest problem, with Nigeria and South Africa topping the list of affected countries. The GFI warns that these illicit outflows have a devastating effect on African economic development and stability. The G20 group of the world’s leading economies is addressing this by automatically exchanging tax information to capture tax dodgers and block the transfer of illegal money. (*Thomson Reuters Foundation*, 12 Dec 2013)

• The UN appealed for a record US$ 6.5 billion for Syria and its neighbours, to help the 16 million victims of the 33–month conflict. The appeal covers food, drinking water, shelter, education, health and polio vaccines. More than 100 000 people have been killed, millions displaced, cities have been destroyed and the economy wrecked. Destruction of the water network leaves 10 million people relying on the UN to chlorinate water, and energy supplies are severely disrupted. The UN was cautious about a peace break–through, and stated that even if the violence stops immediately there will be an ongoing need for humanitarian assistance. (*Reuters,* 16 Dec 2013)

• Dr Anita Zaidi, a paediatrician from Pakistan, beat 550 entries to win the first US$ 1 million Caplow Children’s Prize. She will use the prize money to help residents of Karachi’s Rehri Goth district, whose residents are so poor and isolated from medical services than 11% of children die before the age of 5. She will train midwives and set up transportation networks to take mothers in birth crises to hospital. She will address local prejudices against hospitals, and distribute food, vitamins and vaccines. The prize was endowed by Ted Caplow, an entrepreneur whose premature triplets spent a month in intensive care. (*New York Times*, 30 Dec 2013)

• Extreme poverty and a harsh climate means that one–third of Afghan people have insufficient food to lead active, healthy lives, and another third risk food insecurity. Food shortages are particularly damaging to children; more than 50% of Afghan children suffer cognitive and physical damage due to malnourishment in the first two years of life. A UN study found that even a minimally health diet is beyond the reach of the majority of Afghans. To help tackle this, the government has begun fortifying certain foodstuffs with micronutrients. Adequately–fed children can earn between 33–50% more as adults compared to their malnourished peers, and malnutrition reduces Afghanistan’s national income by 2–3% annually – a loss of US$ 500 million to an impoverished country. (*The Guardian*, 26 Jan 2014)

• On his first visit to Myanmar, the World Bank’s President Jim Yong Kim announced a US$ 2 billion development program for the country. It will include projects to improve access to energy and health care for poor people, and support other key development priorities. As over 70% of Myanmar’s people lack access to reliable electricity supplies, US$ 1 billion is earmarked to expand electricity generation. Another US$ 200 million is set aside to help achieve universal health coverage by 2030. An estimated 75% of Myanmar’s mostly rural population lacks access to quality health care, and high costs place most essential services out of the reach of many families below the poverty line. (*World Bank,* 26 Jan 2014)

## AUSTRALIA AND WESTERN PACIFIC

• Former Australian Prime Minister, Kevin Rudd, has denied any ambitions to lead the UN as Ban Ki–moon’s successor. Stating that he always intended to remain involved in global politics, he would not comment on domestic issues. In a speech to The International Institute for Strategic Studies in London, he outlined the repositioning of global power towards China, describing its rise and the impact of international order as “the great challenge of our time.” (*The Guardian,* 17 Dec 2013)

• Dengue virus serotype 3 recently re–emerged in several countries and territories in the South Pacific, including Fiji, French Polynesia and Kiribati, after nearly 20 years’ absence. There are four strains of the dengue virus, and infection with one strain will provide immunity against it but not the others. Dengue serotypes can re–emerge after absences of 15–20 years as children who are growing up without exposure to it create a susceptible cohort. Dengue is transmitted by mosquito bite, and there is no vaccine or specific treatments. Mortality rates are low provided that it is recognised early and appropriate care is sought. Affected areas are taking action by surveillance, controlling mosquito numbers and clinical care. (*WHO,* 16 Jan 2014)

• Australia’s federal government will cut Australia’s contribution to tackling climate change, health and sanitation crises in developing countries as part of its US$ 610 million cuts to foreign aid. The timing, well into the 2013–14 financial year, is described as almost as damaging as the cuts, because funding is withdrawn from initiatives that are already underway. The aid agency Oxfam says it must now consider which critical work it can no longer do. Australia will cut funding to humanitarian, emergency and refugee programs, plus contributions to the UN, Commonwealth and other international organizations eg, WHO and UNICEF. Australia will no longer commit to a timeline for contributing 0.5% of its GNP to overseas aid – part of its MDG commitment. (*Brisbane Times*, 18 Jan 2014)

• Former Australian Prime Minister Julia Gillard is the new head of the Global Partnership for Education, which works towards getting 57 million children into school and has allocated more than US$ 2.8 billion to education. Ms Gillard is concerned about the 6.3% fall in aid spending for basic education from 2009 to 2011, as the latest UN figures show 57 million children are not in school, and 250 million children lack basic numeracy and literacy skills. She hopes that Malala Yousafzai, the UN Special Envoy for Education shot by the Taliban for demanding girls’ education, will raise the profile of the global state of education. (*Sydney Morning Herald,* 11 Feb 2014)

• A study shows that Australia’s mass human papillomavirus (HPV) vaccination program is working, and fully vaccinated women are much less likely to develop cervical cancer. HPV can also cause penile, anal, cervical, vulvar and vaginal cancers, and genital warts. The vaccine halves the risk of cervical cancer by preventing infection by two types of HPV, saving lives and minimizing future health expenditure. However, regular smear tests are still vital, as the vaccine does not protect against other HPV strains. (*The Guardian*, 4 Mar 2014)

## CHINA

• China’s air pollution was the worst for 52 years, with 13 provinces hitting record high levels and nearly half of China being affected by smog. There are increased efforts by government, laboratories and universities to work collaboratively in understanding the causes of air pollution and how it is dispersed or concentrated in the atmosphere. China’s five year action plan aims to improve technology, planning and regulation, and emphasises making polluters pay, rewarding energy efficiency, conservation, and reduction efforts. China is applying existing technologies more extensively, leading to cleaner emissions from power stations, cleaner heating systems and more recycling of agricultural waste. Air pollution is a serious health risk but there are intensive efforts to improve air quality, with funds to subsidise environmentally–friendly industries, improved policies on pricing and taxation, and encouraging investment in air pollution control technology. (*The Guardian,* 18 Dec 2013)

• China is expected to become the world’s second–largest pharmaceutical market by 2016, fuelled by an ageing population, expanding public health insurance and the increasing demands of a wealthier society. However, even cheap generic drugs are much more expensive than their international benchmark. This is caused by doctors being underpaid, leading to them making up their income by drug prescriptions; and hospitals, with few available revenue streams, can charge a 15% mark–up on medicines. Medicines now comprise 40% of total health expenditure, compared to the 16% OECD average. Promoting the use of cheaper generic drugs is difficult, partly because scandals involving unsafe food and drugs make many patients and doctors favour expensive foreign–brand drugs. Efforts to promote cheaper drugs by requiring hospitals to buy them through bidding have largely failed, and indeed may have caused a shortage of some life–saving drugs. (*The Economist,* 1 Feb 2014)

• Smog is not the only deadly air pollutant in China, as 300 million adults smoke and 700 million people are exposed to passive smoking. China is the world’s largest tobacco market and an estimated 100 million people will die from smoking–related illnesses this century. This has led to the government taking some measures, including a partial smoking ban. However, these have had little impact, and studies show that tobacco damage is set to rise. Even the proposed beefed–up measures are inadequate, and the tobacco industry is very closely linked with government. If proper anti–smoking measures (eg, health education, heavy taxation, widespread smoking bans, quitting schemes etc.) were in place, an estimated 13 million lives would be saved, and 154 million “life years” gained. The industry is largely self–regulated and its tax revenue support government finances, making more stringent actions unlikely. (*The Economist,* 1 Mar 2014)

• China, with more than 10% of all tuberculosis (TB) cases, is a major contributor to the global TB pandemic. However, it has halved its TB prevalence, with rates falling from 170 to 59 per 100 000, and the WHO says other countries could use a similar approach. Between 1990 and 2000, TB levels fell where the WHO–recommended programme of rapid detection and treatment was implemented, and by 2010 prevalence fell by 57%, tripling this reduction. The 2014 World Health Assembly will look at setting new targets for prevalence reduction and TB elimination. However, nearly 4000 people die each day from TB, and 3 million cases go undiagnosed each year. (*BBC News*, 18 March 2014)

• China recently set up the alliance of Organ Procurement Organisations (OPO), to improvement the management of organ donations. The OPO chairman, Huang Jiefu, has been progressing reform in the nation’s organ donation and procurement systems. Each year, approximately 300 000 people need an organ transplant, but only 10 000 are carried out due to a shortage of donors. Across China, only 169 hospitals are authorized to carry out transplants. (*Xinhau,* 20 Mar 2014)

## EUROPE

• European countries are accused of profiting from their aid budgets, by increasing the amount given as loans, often with high interest, which are classed as Official Development Assistance (ODA). The European Network on Debt and Development (EURODAD) calls for urgent reform, as these loans cost developing countries US$ 828 million each year, reducing resources for desperately poor people. EURODAD calls for avoiding loans unless they have a positive impact, and donors should not be incentivised to give loans if grants are preferable. (*The Guardian*, 16 Jan 2014)

• The UK will spend US$ 3 billion on the economic development of poor countries in 2015, more than double the amount in 2012–13. It will continue with traditional aid programmes (eg, disaster relief, disease) but will shift future resources towards economic development, particularly growth and jobs. This coherency is a shift from its previously *ad hoc* approach. Examples of the new “smart aid” include providing technical support to improve Nigeria’s power supply, and modernizing Mombasa, east Africa’s largest port. The UN is concerned that high rates of Africa’s economic growth rates have not translated into job creation, and NGOs call for the UK government to ensure that projects to boost trade help, not hurt, poor people. (*The Guardian*, 27 Jan 2014)

• In Scotland, male alcohol–related deaths are double that of the rest of the UK, the cirrhosis mortality rate is one of the highest in Western Europe, and alcohol–related problems cost US$ 1.7 billion a year. Between 1980 and 2005, alcohol became 62% more affordable with a corresponding rise in consumption, thus making a strong case for price intervention. In 2012, the Alcohol Minimum Pricing (Scotland) bill was passed by the Scottish Parliament, but was immediately challenged by the Scotch Whisky Association. Meanwhile, the bill is in limbo until the ongoing legal disputes are resolved. (*BMJ,* 6 Feb 2014)

• Analysis by the Institute of Cancer Research (ICR) and Innovative Therapies for Children with Cancer shows that children with cancer are denied new, potentially life–saving, drugs because EU rules allow companies to trial some drugs only in adults. Drug companies can gain exemption from testing in under–18s, even if the drug may work in children, if the adult cancer does not occur in children. However, many modern drugs are targeted at genetic features of the cancer that can be common to different types of adult and child cancer. This causes delays in drugs becoming available for children, and some may never be licensed. The ICR calls for urgent reform to enable more drug–testing in children, and for more financial incentives for drug companies to develop drugs for small patient populations. *(Institute of Cancer Research,* 10 Feb 2014)

• The International Monetary Fund is near to agreement with Ukraine on an aid package worth US$ 14–18 billion over the next two years, which will potentially unlock a further US$ 10 billion of loans from the EU and the US. It is tied to an economic reform program, including cuts to energy subsidies that could see a 50% increase in domestic gas prices, and restructuring the state–owned energy company, whose deficit is nearly 2% of GDP. Ukraine’s new Prime Minister warned that the economy could contract by up to 10% in 2014 without these austerity measures. The IMF will review Ukraine’s anti–corruption, tax and legal frameworks – currently it is listed at 144 out of 177 countries in an international ranking of corruption perception. (*BBC News,* 27 Mar 2014)

## INDIA

• Despite its reputation for being unwelcoming to foreign businesses, international fast–food chains are being welcomed in India by a young, upwardly mobile population. The spending power of this group is rapidly increasing, as more people, particularly women, enter the workforce and people acquire new tastes. The Indian market for chain restaurants is an estimated US$ 2.5 billion in 2013 and expected to grow to US$ 8 billion by 2020, driven by the growth of fast–food restaurants. However, health experts are concerned about the impact of public health, although businesses have encountered little public opposition as they are not perceived as replacing traditional eateries. They all face challenges of adapting their products to local customer needs without compromising their core product. (*New York Times*, 8 Jan 2014)

• India has reached a major milestone in the eradication of polio as its last recorded case occurred three years ago, putting it on course to be polio–free by March 2014. The fight against polio was made more difficult by the problems of poverty, dense population, poor sanitation, high levels of migration and a weak public health system. In 2012, the WHO declared India free from active endemic wild polio transmission. The victory against polio is India’s second major health achievement, after the elimination of smallpox in 1980. However, there are concerns that polio may re–enter India from Pakistan, where cases have been reported. (*The Guardian,* 13 Jan 2014)

• India launched its national adolescent health strategy with the support of the UN Population Fund. A fifth of India’s population is aged 10–19, so the benefits of a healthier youth will have a profound impact on the entire population, and is an investment in the future workforce, parents and leaders. The strategy will provide health, information and services aimed at adolescents, including girls and marginalised groups. Investing in this group could result in a demographic dividend – the accelerated economic growth that can result from a rapid decline in a country’s fertility rate coupled with smart investments in health, education and job creation. (*UN Population Fund,* 17 Jan 2014)

• India’s 2014 general election will be the largest democratic event in history, with more than 814 million people entitled to vote to decide the country’s 16th government. Polling begins on 7 Apr, and ends on 12 May with the result decided by 16 May. The election’s sheer scale is unprecedented; in nine polling days spread across five weeks, the world’s largest electorate will visit 930 000 polling booths to cast their votes using 1.7 million electronic voting machines. For the first time, voters can select “none of the above”, allowing them to reject parliamentary candidates. The impact of the youth vote and technology will be scrutinised: 24 million 18–19-year olds will participate in an election where social media and internet campaigning have featured heavily. Campaigning costs are high too, as the incumbent government faces a closely–run election and possible defeat. (*The Diplomat*, 13 Mar 2014)

• Six Indian innovators were selected to contribute to the development of sanitation solutions as part of the Reinvent the Toilet Challenge (RTTC): India. This India–specific program is modeled on the Gates Foundation’s global RTTC, and is a collaborative effort to develop innovative, safe and affordable sanitation technologies, and to drive research, development and production of “next generation toilets.” The grants were announced at the event fair, which was co–hosted by the Indian Government and the Gates Foundation. The fair was an opportunity for the 16 RITTC grant–holders to show–case progress to date and their project prototypes. (*BMGF,* 22 Mar 2014)

## THE AMERICAS

• The Canadian Supreme Court unanimously struck down the country’s anti–prostitution laws, following a challenge brought by current and former sex workers. Prior to this, selling sex was not illegal, unlike brothel keeping, soliciting, or living off the earnings from sex work. In striking down the law, the Court recognized that its provisions prevented people engaged in a risky, but legal, activity from taking steps to protect themselves. The decision gives the Canadian Government one year to devise new legislation on sex work. (*BBC News*, 20 Dec 2013)

• Nicaragua is the first country to ratify the Protocol to Eliminate Illicit Trade in Tobacco, the world’s first international public health treaty. It aims to eliminate all forms of illicit trade in tobacco, and to co–operate internationally on this issue. Colombia, Costa Rica, Ecuador, Panama and Uruguay have also signed the protocol and are expected to ratify it soon. It must be signed by 40 countries before it can enter into force. An estimated 10% of the global cigarette trade is illicit, posing serious public health risks as it makes tobacco cheaper and more appealing to vulnerable groups such as youngsters and poorer people. It also causes revenue losses to governments. In the Americas, 16% of deaths amongst people aged 30 years and older is attributable to smoking; the joint highest in the world alongside Europe. (*PAHO,* 16 Jan 2014)

• The last reported case of endemic transmission of measles in the Americas was in 2002, and measles deaths have disappeared from the region. This makes the Americas the first region globally to eliminate measles – a leading cause of death for young children. Key reasons include high vaccination coverage and the early detection of cases. The WHO/PAHO measles elimination strategy was based on experience gained in polio eradication: national vaccination ‘catch–up’ programmes targeted at children; strengthening routine immunisation services; and mass follow–up campaigns. However, there are imported cases so vigilance is needed to avoid reintroduction and any outbreaks of the disease. (*PAHO,* 10 Feb 2014)

• There was a reported 43% drop in obesity rates amongst old children 2–5 years old in the USA; the first indications that obesity trends in America’s youngest children may be turning a corner. Approximately 8% of children in this group were obese in 2012, down from 14% in 2004. It is the first evidence of any obesity declines amongst any age group, and bodes well for the future as obesity becomes established at this age, and can be very difficult to shake off in later years. However, this is still a very small percentage of the American population, and obesity rates in the rest of the population have remained constant; even increasing for women aged over 60. Possible reasons for the decline include less consumption of sugary drinks, higher rates of breastfeeding, less calorie consumption amongst children, and the impact of anti–obesity programmes. (*New York Times*, 25 Feb 2014)

• The end of March 2014 was the deadline to sign up for health insurance under the US Affordable Care Act, also known as “Obamacare”. It will be some time before it is known how many people have signed up for coverage, but already some things are clear. First, more people have coverage in part due to the 26 states that have expanded Medicaid, and thus covering millions of poor adults. However, coverage is far from universal with half of all states not expanding Medicaid coverage, and serious glitches in the online health insurance system has led to enrolment problems. The importance of young, fit people purchasing insurance to help pool risk may be over–stated, as Obamacare contains mechanisms to smooth risk until 2017, and companies have limited time to set their rates for 2015. (*The Economist,* 31 Mar 2014)

